# The Cause of Quackery in English Dentistry

**Published:** 1908-03-15

**Authors:** B. S. Grant

**Affiliations:** Glasgow


### THE CAUSE OF QUACKERY IN ENGLISH DENTISTRY.

BY R. S. GRANT, L.D.S., GLASGOW.

First of all, let us consider what is the cause of quackery. I believe it is mainly caused by our method of teaching the mechanical or prosthetic branch of our work. Our system of taking apprentices to serve for a number of years is in a great measure responsible for the present condition of matters. Let us seriously consider the question whether this is so or not.

Do we not take into our workrooms as apprentices boys whom we feel will never be able to pass the preliminary exam. in general knowledge, let alone qualify, and yet we go on training them? If they are not able to pass this exam. they are not fit to become professional men. The apprentice system is no doubt suitable for the training of tradesmen, and when we adopt their methods can we wonder if we bring up a race of tradesmen?

If our object is to produce professional men, with professional ideas, and a soul above commercialism and mere money making, we must first select suitable recruits, and bring them up in an atmosphere of professionalism. At present we bring them up as tradesmen, and they have neither the education nor ability to get out of it.

When they have learned what they consider enough from us they join the ranks of the unregistered practitioners, where they soon learn of our inability to put them down, and also the disinclination of Parliament to frame laws for their extinction. In a word, if we allow them to enter our profession we cannot put them out and we, as their teachers, are wholly responsible.

Let us now consider what is the remedy for the muddle we have got into through the fault of our predecessors and of ourselves.

Let us agree to teach only boys who have passed the prelim. Insist on receiving a premium, or as an equivalent to serve longer time, but let them understand that they are being taught, that they are not working for wages.

The question of whether mechanics should be taught privately or in college I do not intend to enter into, but I fully believe if we did do away with private tuition, and had all work taught at a school, more men would qualify proportionately and quackery would decrease.

When I was permitted to enter the profession, there was more of a professional spirit among apprentices; each one thought that he must qualify, or be a mechanical assistant all his life. But now things have changed. Many enter the profession—and we make it easy for them to do so—with no intention to qualify; others try in a half-hearted way to pass the prelim. but after one or two feeble attempts give it up, and with it all hope of qualifying.

It is not only they whom we lose, but to carry on their trade, or business, or whatever you like to call it, they train others, and so it will go on until they become so powerful that they will swamp us. They are so powerful even now that we find it impossible to put them down, but we can at least prevent their accumulation.

It may seem to you that I am closing the doors to a suitable youth who possesses the ability and spirit to become an honour to our profession, but who lacks the wherewithal.

But as an Association we could assist any deserving youth, if he showed brilliancy. If he passed the prelim. with distinction we could assist him during his mechanical instruction. If he passed the professional exam. in mechanics, well we could help him further, and he would be in a position to earn money as a mechanical assistant. It would be better to help him than allow him to go over to the quacks, and then spend the money that would have helped him in trying to put him down. What I mean is to give assistance in the form of bursaries to brilliant students in need of help.

Let us look into the future, and see what will be the position of mechanical dentistry. Every year the proportion of dental students taking the medical degree is increasing. With extra lectures and other work required they have less time for mechanics; consequently it is neglected in favour of their other work.

They are being encouraged in this by the compulsory time in mechanical training being shortened.

As these men have not had sufficient training in prosthetic work, when they start practice they will either throw it over or employ mechanics to do it for them. They will also train apprentices under these assistants. As time goes on the mechanics will not work for a specialist for a weekly wage when they can make more money working to the profession generally, or to the public direct. In that case the specialist will give up this branch altogether.

Eventually there will be two distinct classes of workers. The mouth-specialist who will have a medical degree, and the artificial denture maker, who will have learned that branch only. The specialist will do conservative work only, and will send his patients to the mechanic when artificial substitutes are required.—*The British Dental Record.*

